# Free Mercury in Amalgam Fillings

**Published:** 1874-06

**Authors:** H. L. Sage


### FREE MERCURY IN AN AMALGAM FILLING.

BY H. L. SAGE, D. D. S.

In looking over a number of extracted teeth, I discovered a very much discolored left superior molar, with two-thirds of the crown absent, and the palatal wall broken down, leaving only the buccal and a small portion of the grinding surface intact, while that remaining was protected (?) by a large, black, porous and friable amalgam filling extending into the pulp cavity for some distance, but probably not into the roots to any considerable extent, for only a part of the filling has been removed, lest its value as a curiosity should be lessened.

On making an examination, half a dozen or more globules of free mercury, some of them about the size of mustard seeds, were found in the body of the filling.

The history of the case is, to me, unknown, or for whom or when the tooth was extracted; as the peculiar character of the filling was not noticed at the time, and so the tooth was thrown aside with others.

But the filling was evidently inserted by a very neat and expert, not to say conscientious operator. This statement may be received "with a few grains of allowance."

It is to be regretted that nothing is known as to the effects upon the patient of such a finely (?) manipulated specimen of amalgam, or whether or not it was, in this case, innocuous; but it is difficult to imagine how such a filling could be inserted, without some of the mercury escaping during the time required for hardening, or by and during the after wear and tear of mastication, upon such a friable and porous compound, especially, if the tooth had not lost its antagonist.

Again, the temperature of the mouth is 100° F. or nearly, and if mercury evaporates at all temperatures above 66°, as has been stated, more or less of the vapor of this metal would probably be taken up by the lungs and enter the circulation during the process of hardening of the filling, whether in sufficient quantity to prove deleterious or not.

So far as injurious effects are concerned, from an amalgam filling so large, spongy and loose in the aggregation of its particles as was this, and loaded with free mercury at that, the conditions in respect to the filling were favorable for the production of injury, if amalgam, placed in the teeth, is at all pernicious, when manipulated in such wise, or even properly, as Piggott, Harris and others have maintained, contrary to the belief of some others of equal eminence, and of many lesser lights and shadows too, judging by the immense quantities of this material annually sold to dentists in this and other lands.

In closing these observations upon the before mentioned case, the conclusion is irresistible, that, had the filling broken down or worn away to any extent, as it may have done, some of the free mercury must have passed into the stomach; and that, during the hardening of this material, some evaporation of the mercury must have taken place, but with what results can not, as before stated, be known.

"Poisoned by mercury from a tooth filling" was the verdict of a famous western jury. Certainly, if a patient were susceptible to the action of mercury, employed in such a careless manner, the "cause" was present in this case, whether followed by the "effect" or not.

J. Payne, D. D. S., in the Chicago Medical Journal, advances the theory, that many cases of poisoning are produced by the employment of amalgam for fillings in teeth, and that of more than twenty cases which he has had, most of that number had been pronounced consumptive by some physician; the symptoms being a very bad cough, sunken eyes, haggard expression, deep blue or dark color under the eyes, a metallic taste in the mouth, copious flow of saliva, extreme prostration, etc.

The patient, he says, "is liable to be treated for dyspepsia, neuralgia, paralysis, consumption and numerous throat diseases." These effects are produced, he states, by the formation of corrosive sublimate in the mouth; the quick-silver in the plugs being driven off in minute quantities by the heat of the mouth and combining with the chlorine in the fluids thereof, or any saline substance, passes into the stomach and produces slow poisoning.

Perhaps the profession will be slow to believe that these effects are due to amalgam poisoning, without the evidence is more conclusive than that presented by this writer.

But in regard to the pernicious effects of amalgam fillings, it is evident that they may be lessened or entirely prevented, and that, in many cases, a larger quantity of mercury is left in the plug than is necessary.

Of course, the more plastic such a filling is, up to a certain point, the more easily it is introduced into the cavity. But ease of manipulation should be a secondary consideration. But if the mercury is forced out, so as to leave only just enough of it in the compound to unite it by dint of careful packing, it will produce a harder and more durable filling, a filling less liable to contract or change color, and at the same time be much less objectionable on the ground of its pernicious effects on the system.

